# Oncological right hemicolectomy in a trimodal comparison: open surgery versus laparoscopic procedures with extra- and intracorporeal anastomosis technique

**DOI:** 10.1007/s00384-023-04587-3

**Published:** 2024-01-03

**Authors:** Kerstin M. Kerber, Svetlana Hetjens, Christoph Reißfelder, Florian Herrle, Steffen Seyfried

**Affiliations:** 1 Department of Surgery, Universitätsmedizin Mannheim, Medical Faculty Mannheim, Heidelberg University, Mannheim, Germany; 2 Department of Medical Statistics and Biomathematics, Medical Faculty Mannheim, University Heidelberg, Mannheim, Germany

**Keywords:** ERAS, Colon cancer, Extracorporeal, GIQLI, Recovery

## Abstract

**Purpose:**

This study aimed to investigate the surgical short- and mid-term outcomes, as well as the impact on quality of life and recovery, following oncological right hemicolectomy. To accomplish this, three patient cohorts were examined, which included laparotomy OA), laparoscopy with intracorporeal anastomosis (LIA), and laparoscopy with extracorporeal anastomosis (LEA). Our hypothesis was that the group undergoing intracorporeal anastomosis would demonstrate superior outcomes compared to the other cohorts.

**Methods:**

The analysis included a total of 135 patients who were enrolled between 2015 and 2020. In addition to retrospectively collected data, we conducted follow-up surveys using a validated Gastrointestinal Quality of Life Index (GIQLI) questionnaire and semi-structured interviews. These surveys were conducted between July and September 2021 to gather comprehensive information regarding the patients’ quality of life.

**Results:**

The study cohort was divided into OA (*n* = 67), LEA (*n* = 14), and LIA (*n* = 54). The duration of surgery was significantly longer in the laparoscopic groups (median = 200.5 (LEA) and 184.0 (LIA) min vs 170.0 min (OA); *p* = 0.007), while the length of hospital stay was significantly shorter (median = 6.0 and 7.0 days vs 9.0 days; *p* = 0.005). The overall postoperative complication rate was significantly higher in the laparotomy group compared to the intracorporeal group (64.2% vs 35.2%; *p* = 0.006), with the extracorporeal group having a rate of 42.9%. Reoperation within 30 days occurred exclusively in the open surgery group (*n* = 9; 13.43%; *p* = 0.007). The overall response rate to the survey was 75%. Overall, the GIQLI score was comparable among the three groups, and there were no significant differences in the questions related to recovery, regained function, and contentment.

**Conclusion:**

The laparoscopic approaches demonstrated significantly lower complication rates compared to laparotomy, while no significant differences were observed between the two laparoscopic techniques.

## Introduction

Right hemicolectomy with complete mesocolic excision is a common surgical procedure. While the open surgical approach was initially the preferred method, laparoscopic variants have become established due to their advantages in terms of early recovery and short-term complications [1–3]. The minimal invasive approaches are advocated especially within the context of Enhanced Recovery after Surgery (ERAS) Protocols [4, 5]. In some studies, the intracorporeal anastomosis technique (LIA) offers explicitly better outcomes compared with the extracorporeal technique (LEA). However, in other cases, the results of the individual parameters differ from one another [6–8]. Therefore, the optimal anastomotic approach (LIA versus LEA) in right hemicolectomy still remains debatable, and further research is needed to determine the preferable approach. The majority of existing studies deal exclusively with short-term results for the comparison of surgical methods. However, we have expanded the scope in this study by including a patient survey, thereby additionally measuring functional and mid-term therapy outcomes.

Our hypothesis was that LIA may be superior to the other two groups (open right hemicolectomy (OA) and LEA) regarding short-term, long-term, and functional outcomes. This theory is based on the potential advantages of the intracorporal anastomotic technique such as reduced traction on the mesentery, which can lead to faster recovery and fewer complications [9–11].

## Material and methods

The present study was a non-interventional, monocentric study that utilized a retrospective identification process of patients from our prospectively maintained colorectal cancer patient registry followed by the collection of follow-up data through questionnaires.

We conducted a retrospective analysis of all consecutive patients who underwent an oncological right hemicolectomy at the Mannheim University Hospital between 01/2015 and 08/2020.

The study population consisted of adult patients who underwent an elective right hemicolectomy at our tertiary care center for colon cancer. Patients who underwent converted surgery were categorized as belonging to the open procedure group, while patients with preservation of a stoma were excluded from the study. Furthermore, patients with distant metastases or other surgical procedures within the same intervention were also excluded.

Throughout the data collection period, all patients were managed using a standardized patient care pathway and operated on by the same surgical team consisting of three surgeons.

The aim of the study was to compare three patient cohorts with regard to short- and mid-term, as well as functional outcomes. Short-term outcomes were perioperative outcomes and complications occurring up to 30 days (or during hospitalization if longer than 30 days) postoperatively.

The study assessed patient-reported functional outcomes and mid-term therapy outcomes through a questionnaire and telephone interview, respectively.

For the survey, the validated GIQLI = Gastrointestinal Quality of Life Index was used [12].

### Statistical analysis

For descriptive statistics, the program SPSS version 24.0.0.0 (SPSS Inc., Chicago, IL, USA) and, for further statistical analysis, the program SAS 9.4 (SAS Institute Inc., Cary, NC, USA) were used. Tests for comparing two qualitative groups were the chi^2^-test or Fisher’s exact test. If two quantitative groups were compared, then the *t*-test was used for normally distributed parameters, and the *U*-test was used for non-normally distributed parameters. When comparing more than two quantitative groups, ANOVA was applied if the data were normally distributed, and the Kruskal–Wallis test if the data were not normally distributed. Normal distribution was tested using the Shapiro–Wilk test. The significance level was set at *p* < 0.05. The test according to De Long has been applied for comparing the areas under the receiver operating characteristic (ROC) curves (AUC).

## Results

**Fig. 1:**
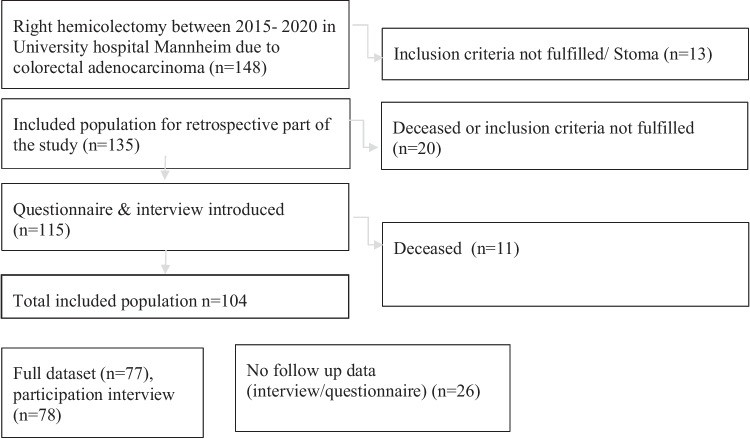
Patient inclusion flow chart

A total of 148 eligible patients were identified and included in the retrospective analysis, and ultimately 115 datasets were included for follow-up data collection, resulting in a response rate of 75% as demonstrated by the inclusion flow chart (Fig. 1).

There were no significant differences between the three groups in the baseline characteristics (Table 1).

**Table 1:** Comparison of three patient cohorts in the baseline characteristics

	Laparotomy open surgery (*n* = 67)	Laparoscopy, extracorporeal anastomosis (*n* = 14)	Laparoscopy, intracorporeal anastomosis (*n* = 54)	*p*-value
Mean age at time of surgery (years) (SD)	69.18 (11.1)	70.21 (7.6)	71.46 (8.8)	0.4559
Gender				0.7431
Male, *n* (%)	31 (46.3)	8 (57.1)	25 (46.3)	
Female, *n* (%)	36 (53.7)	6 (42.9)	29 (53.7)	
Mean BMI (kg/m^2^) (SD)	28.2 (6.2)	28 (6.7)	26.9 (5.3)	0.6391
Median ASA (IQR)	2 (1)	2 (1)	2 (1)	0.6571
Median tumor stage UICC (IQR)	2 (2)	3 (2)	2 (1)	0.5919
Chemotherapy treatment performed, *n* (%)	14 (29.8)	2 (18.2)	11 (29.7)	0.7114
Patients with previous abdominal surgery, *n* (%)	39 (58.2)	7 (50.0)	23 (42.6)	0.2315
Any chronic disease, *n* (%)	57 (85.1)	11 (78.6)	46 (85.2)	0.8145

The following anastomosis techniques were used (distinction stapled as end-to-end anastomosis versus handsewn with side-to-side anastomosis):

54 anastomoses were handsewn (80.6%) and 13 were stapled (19.4) in the OA group; in the LIA group, 12 anastomoses were handsewn (85.7%) and 2 were stapled (14.3%). In the LIA group, all 54 anastomoses were performed by stapler (100.0%). There was a significant difference between the anastomosis techniques used (*p* < 0.0001).

The duration of surgery was significantly longer in the LIA compared to the OA (*p* = 0.007). In contrast, the length of hospital stay was significantly shorter in both laparoscopic procedures compared to the open technique (*p* = 0.005) (Table 2).

**Table 2:** Comparison of three patient cohorts regarding the length of hospital stay

	Laparotomy open surgery, *n* = 67	Laparoscopy, extracorporeal anastomosis, *n* = 14	Laparoscopy, intracorporeal anastomosis, *n* = 54	*p*-value
Surgery time, median (IQR)	170 (60)	184 (50)	201 (79)	0.0069^*^
Length of hospital stay in days, median (IQR)	9 (8)	7 (2)	6 (3)	< 0.0001^*^
Comprehensive complication rate (any complication), *n* (%)	43 (64.2)	6 (42.9)	19 (35.2)	0.0055
Wound infection rate (SSI), *n* (%)	9 (13.4)	1 (7.1)	6 (11.1)	0.7843
Anastomotic leakage, *n* (%)	4 (6.0)	0 (0.0)	0 (0.0)	0.2121
Incisional hernia, *n* (%)	5 (7.5)	1 (7.1)	0 (0.0)	0.0687
Ileus, *n* (%)	1 (1.49)	0 (0.0)	0 (0.0)	1.0000
Paralysis	16 (23.9)	3 (21.43)	3 (5.6)	0.0217^*^
Reoperation within 30 days post-surgery	9 (13.4)	0 (0.0)	0 (0.0)	0.0070
Regular intestinal passage, *n* (%)	46 (78.0)	10 (76.9)	44 (91.7)	0.1348
Regular oral intake, *n* (%)	51 (86.4)	12 (92.3)	45 (95.7)	0.2976
Primary wound healing, *n* (%)	52 (88.1)	12 (92.3)	44 (91.7)	0.9049

### Postoperative outcomes (comparison of three patient cohorts)

Postoperative complications occurred significantly less frequently in the intracorporeal technique group compared with laparotomy. Postoperative complications included: postoperative bleeding, intra-abdominal infections, surgical site infections, ileus, anastomotic leakage, incisional hernia, deep vein thrombosis, cardiac and pulmonary complications, renal failure, intestinal atonia (also recorded if transient and present from the first day postoperatively), other complications such as postoperative delirium or electrolyte imbalance.

Another significant difference in favor of the intracorporeal technique versus open procedure represented the occurrence of postoperative intestinal atony (5.6% vs 23.9%; *p* = 0.0217).

Reoperations within 30 days due to the original procedure (right hemicolectomy) occurred only in the laparotomy group (13.4% vs 0.0%; *p* = 0.0070) and were significantly more frequent than in the two laparoscopy groups. Major complications occurred exclusively in the open surgery group, as indicated by the necessity for reoperation (Clavien-Dindo Grade IV). This was primarily attributable to anastomotic insufficiency in four cases, requiring surgical revision. Additionally, there were three instances of reoperation involving deep infection necessitating operative wound revision under anesthesia. In one case, reoperation within 30 days was imperative due to an incarcerated incisional hernia. Four patients underwent initial laparoscopic surgery and were ultimately converted. The analysis was subsequently conducted within the framework of the open collective. One patient from the converted group experienced an anastomotic insufficiency, subsequently developing a wound infection, thus contributing to the complications of the cohort.

The other postoperative outcomes showed no significant differences between the three groups.

### Mid-term and functional outcomes

To determine whether the interviewees were representative of the overall population of patients, direct comparisons of baseline data, perioperative, and postoperative therapy outcomes were made between the group of responders and non-responders. The groups could be considered comparable to each other, and thus the interview and questionnaire results are considered representative of the overall population.

Of all included patients in the study (*n* = 135) 77% (*n* = 104) could be contacted. Of these, 78 (75%) patients participated in the interview and questionnaire. It should be noted in the survey that the time interval from surgery to interview differed between the groups: the median in OA was 51 months (minimum 12; maximum 72) and in LEA 53.5 months (minimum 42; maximum 68). This results in a significant difference from LIA, in which the median value was 16 months (minimum 8; maximum 38), *p* < 0.0001.

The results of the GIQLI survey did not show any significant differences between the three patient cohorts nor in the comparison of the subtotal or the total. The only exception was a single question on disturbing changes in outer appearance (*p* = 0.0387), but a detailed analysis of this single question was not performed.

The highest mean scores in the symptoms and emotions categories were achieved by LEA, as well as the highest total score (112.2 vs. LIA 112.0 vs. 110.75). In the physical functions category, LIA showed the highest score, as well as the highest mean score per item with a very small margin over LEA (LIA 3.1399 vs LEA 3.1384 vs OA 3.0863). In the category of social functions, OA had the highest score (Table 3).

**Table 3:** Symptoms and emotions categories

	**OA**	**LEA**	**LIA**	**Total**	***p*-value**
**Symptoms, mean (SD)**	3.23 (0.54)	3.34 (0.57)	3.31 (0.50)	3.29 (0.52)	0.7460
**Emotions, mean (SD)**	3.19 (0.86)	3.24 (0.98)	3.21 (0.79)	3.21 (0.83)	0.8407
**Physical functions, mean (SD)**	2.49 (1.02)	2.47 (1.01)	2.61 (0.72)	2.55 (0.87)	0.9730
**Social functions, mean (SD)**	3.22 (1.05)	3.08 (1.07)	3.11 (0.83)	3.15 (0.94)	0.4723
**Global score, mean (SD)**	110.75 (24.00)	112.20 (24.47)	112.01 (19.57)	111.6 (21.68)	0.9896
**Average score per single item, mean (SD)**	3.0863 (0.66)	3.1384 (0.66)	3.1399 (0.54)	3.1194 (0.60)	0.9687

In all five questions regarding recovery issues, LEA scored with the highest mean score compared with the other two groups. Both laparoscopic groups considered together also showed a higher mean score in each category compared to OA. LIA showed a lower mean score compared to OA in only one of the five topics (enjoying life) (Table 4).

**Table 4:** GIQLI recovery questions

	OA (*n* = 29)	Laparoscopic surgeries in total	LEA (*n* = 22)	LIA (*n* = 25)	*p*-value
	Mean (SD)	Mean (SD)	Mean (SD)	Mean (SD)	
Returning to habits and routines	3.97 (1.43)	4.63 (0.78)	4.7 (0.48)	4.62 (0.85)	0.1356
Resolution of symptoms	3.97 (1.16)	4.24 (1.23)	4.6 (0.97)	4.15 (1.29)	0.4303
Overcoming mental strains	4.10 (1.23)	4.37 (1.09)	4.4 (1.26)	4.36 (1.06)	0.5994
Regaining independence	4.55 (0.99)	4.69 (0.89)	5.0 (0.00)	4.62 (0.99)	0.3139
Enjoying life	4.38 (1.12)	4.39 (1.11)	4.7 (1.08)	4.31 (1.13)	0.7126

Logistic regression showed the following parameters:

Between the two laparoscopic groups, intestinal atonia emerged as the decisive parameter, in favor of the intracorporeal anastomosis group.

## Discussion

Significant advantages in terms of short-term complications and a reduced length of hospital stay have been extensively documented for the intracorporeal technique. The use of intracorporal anastomosis is associated with a faster recovery of bowel function in some publications.

Our original hypothesis that the technique of intracorporeal anastomosis would be superior to the other two groups could not be confirmed in this study, but neither could it be clearly refuted. Therefore, it remains questionable whether intracorporeal anastomosis should remain the method of choice and whether the complete replacement of the practice of extracorporeal anastomosis is thus finally justified.

After the first report on laparoscopic hemicolectomy in 1991 [13], quite a few studies in the following years showed the advantages and disadvantages of laparoscopic versus open right hemicolectomy based on short- and long-term therapeutic outcomes [1].

The data of our study showed significant differences between the groups of the conventional open procedure and the current best practice technique, laparoscopy with intracorporeal anastomosis. The advantages and disadvantages of short-term therapeutic outcomes of laparoscopic procedures compared with open surgery have already been investigated and described in various studies and include the classic parameters from existing patient data [14–16]. In our study, the significant differences relate to intracorporeal technique associated with shorter hospital length of stay compared with laparotomy, as well as shorter complication duration, and decreased incidence of intestinal atony, while also associated with longer operative time. These results are consistent with the findings of other studies [11].

The lower complication rate and decreased incidence of intestinal atony may be explained by a less invasive nature of the intracorporeal procedure by avoiding pulling of the small bowel and colonic mesentery. Moreover, the selection of patients might play a role, as nowadays an open procedure is usually only chosen when laparoscopic surgery is not possible due to difficult access, large T4 tumors, or relevant pre-existing comorbidity. In our study, however, a strong influence on our results could not be confirmed by those variables, and the groups could be considered comparable.

The use of the GIQLI questionnaire [12] was considered appropriate for questioning functional outcomes, as were questions about patient recovery. Surveying the patients themselves does not yet currently appear to be a common method in studies measuring the therapeutic outcomes of surgical procedures. In our view, it is an important tool for detecting improvement areas in patient care and satisfaction.

Regarding the midterm and functional outcomes based on the interview and the GIQLI survey, there were no significant differences between the three groups. None of the three groups was shown to be significantly superior. Comparable studies (using the GIQLI for the same comparison or using the recovery questions in a patient interview setting) could not be found.

In the evaluation of the questionnaire, different groups dominated depending on the category studied. While the superior group in the categories of emotions and symptoms was the group of extracorporeal anastomosis, this superiority for physical functions was shown for the intracorporeal anastomosis technique, and for the category of social functions by the open technique. The best social functions in the group of laparotomy may probably be justified by the longest time interval between surgery and interview allowing patients to adapt to the best possible extent to their social life. It is noteworthy that physical functions showed the highest scores for the intracorporeal technique patient group. Logistic regression showed a similar finding: the parameter that predicts the difference between the two laparoscopic techniques in this case is the parameter of intestinal atonia. This is consistent with the finding of various RCTs attributing the better functional outcomes to intracorporal anastomosis [17–21].

In the interview at the time of patient recovery questions, the extracorporeal anastomosis group performed best in all questions with the highest score. However, it is important to note, as mentioned above, that the time from surgery at the time of the interview was the lowest for the intracorporeal anastomosis group and, accordingly, the time to recovery was the lowest. In addition, it must be assumed that a smaller time interval may have the effect of a more negative evaluation in that the memory and impact of the cancer diagnosis, the surgery, and its consequences are significantly more present. In four of the five questions, the intracorporeal anastomosis group nevertheless performed as the second-best group.

No clear superiority of any of the techniques could be demonstrated in the discussion with the participating patients. However, the laparoscopic techniques appear superior to those of the open procedure in the mid-term outcomes, both for the overall comparison (both laparoscopic procedures versus OS) and for the individual comparison (EA versus OS and IA versus OS).

When comparing the three groups, it was considered that the types of anastomoses used (stapled versus handsewn) may be a possible reason for the short-term differences in the results. Since mainly handsewn anastomoses were performed in the open procedure and extracorporeal anastomosis group, while the intracorporeal anastomosis group was exclusively stapled, this results in an imbalance in the distribution. Therefore, the anastomosis technique cannot be excluded as an influencing factor to explain the existing differences.

## Strengths and limitations

The current study boasts several noteworthy strengths that contribute to its methodological rigor and the valuable insights it offers. These strengths encompass the patient group selection, study design, comparability of groups, response rate, and patient-centered evaluation.

The study’s inclusion of consecutive patient groups undergoing surgical interventions with consistent operative and anastomotic techniques enhances the internal validity of the findings. By minimizing variability in surgical approaches, the study can more effectively isolate and analyze the effects of other variables under investigation. The adoption of a sequential cohort design ensures a balanced distribution of baseline characteristics across the patient groups. This approach promotes meaningful comparisons between cohorts, as the baseline similarities mitigate confounding variables that might otherwise compromise the study’s internal validity.

A significant strength of the study lies in its ability to achieve a high response rate from enrolled patients. Additionally, conducting personal interviews with consenting patients adds depth to the data collection process. These interviews facilitate a comprehensive exploration of patients’ experiences, perspectives, and recovery outcomes, enriching the study’s qualitative insights.

The incorporation of Quality of Recovery (QoR) evaluation distinguishes this study from many others in the field. This patient-centered assessment metric offers a holistic understanding of patients’ postoperative experiences, encompassing physical, psychological, and functional aspects of recovery. By including QoR evaluation, the study contributes to a more comprehensive understanding of patient outcomes and addresses a critical aspect often overlooked in similar research papers.

It must be noted, however, that the small number of cases in the extracorporeal anastomosis technique group in this study makes a comparison with the other two surgical techniques difficult, and thus possible significant differences may remain undetected. The reason for this small number of patients consists of the sequentially changed surgical standard from formerly open surgery via LEA to the standard use of LIA. A larger cohort could provide more clarity here, and a multicenter study would be helpful to reduce this bias.

## Conclusion

In summary, oncological hemicolectomy using laparoscopic techniques confirmed better short-term postoperative results compared with open surgery. No overall superiority of either laparoscopic technique could be detected in our study between intra- and extracorporal anastomotic techniques with regard to quality of life and recovery. However, the LIA intracorporeal anastomosis technique suggests significant advantages for bowel regeneration and reduced bowel atony and may therefore become the current best practice on a larger scale.

## Data Availability

The data that support the findings of this study are not openly available due to reasons of sensitivity and are available from the corresponding author upon reasonable request.
